# Heterogeneous Phenotypic Responses of Antibiotic-Resistant *Salmonella Typhimurium* to Food Preservative-Related Stresses

**DOI:** 10.3390/antibiotics12121702

**Published:** 2023-12-05

**Authors:** Jiseok Yi, Juhee Ahn

**Affiliations:** 1 Department of Biomedical Science, Kangwon National University, Chuncheon 24341, Gangwon, Republic of Korea; leejsdol1008@kangwon.ac.kr; 2 Institute of Bioscience and Biotechnology, Kangwon National University, Chuncheon 24341, Gangwon, Republic of Korea

**Keywords:** *Salmonella Typhimurium*, lactic acid, NaCl, persistence, cross-resistance

## Abstract

This study was designed to evaluate the response of antibiotic-resistant *Salmonella Typhimurium* to food preservative-related stresses, such as lactic acid and sodium chloride (NaCl). *S. Typhimurium* cells were exposed to 1 µg/mL of ciprofloxacin (CIP), 0.2% lactic acid (LA), 6% NaCl, CIP followed by LA (CIP-LA), and CIP followed by NaCl (CIP-NaCl). The untreated *S. Typhimurium* cells were the control (CON). All treatments were as follows: CON, CIP, LA, NaCl, CIP-LA, and CIP-NaCl. The phenotypic heterogeneity was evaluated by measuring the antimicrobial susceptibility, bacterial fluctuation, cell injury, persistence, and cross-resistance. The CIP, CIP-LA, and CIP-NaCl groups were highly resistant to ciprofloxacin, showing MIC values of 0.70, 0.59, and 0.54 µg/mL, respectively, compared to the CON group (0.014 µg/mL). The susceptibility to lactic acid was not changed after exposure to NaCl, while that to NaCl was decreased after exposure to NaCl. The Eagle phenomenon was observed in the CIP, CIP-LA, and CIP-NaCl groups, showing Eagle effect concentrations (EECs) of more than 8 µg/mL. No changes in the MBCs of lactic acid and NaCl were observed in the CIP, LA, and CIP-LA groups, and the EECs of lactic acid and NaCl were not detected in all treatments. The bacterial fluctuation rates of the CIP-LA and CIP-NaCl groups were considerably increased to 33% and 41%, respectively, corresponding to the injured cell proportions of 82% and 89%. CIP-NaCl induced persister cells as high as 2 log cfu/mL. The LA and NaCl treatments decreased the fitness cost. The CIP-NaCl treatment showed positive cross-resistance to erythromycin (ERY) and tetracycline (TET), while the LA and NaCl treatments were collaterally susceptible to chloramphenicol (CHL), ciprofloxacin (CIP), piperacillin (PIP), and TET. The results provide new insight into the fate of antibiotic-resistant *S. Typhimurium* during food processing and preservation.

## 1. Introduction

Since the discovery of antibiotics, they have long been used as successful treatments for bacterial infections [1], but their overuse and misuse has become a major contributing factor to the development of antibiotic resistance in bacteria [2]. Recently, the significant challenge posed by the emergence and spread of antibiotic resistance in healthcare has become a priority in global public health initiatives [3,4]. Bacteria exposed to antibiotic selection pressure can enhance their ability to adapt to unfavorable environmental conditions through various mechanisms and further accelerate the dissemination of antibiotic resistance genes among bacterial populations through horizontal gene transfer [5,6,7]. The mechanisms of antibiotic resistance in bacteria include the enzymatic degradation of antibiotics, the activation of efflux pump systems, alterations in the antibiotic-binding affinity, the modification of membrane permeability, and the use of alternative metabolic pathways [8]. The mobile genetic elements, including plasmids, transposons, integrons, and phages, play a major role in facilitating the horizontal transfer of antibiotic resistance genes [9].

Antibiotic-resistant acquired bacteria can be exposed to various unfavorable conditions, such as food processing- and food preservation-related stresses. Foodborne pathogens undergo phenotypic changes in response to various environmental stresses, including cold, heat, nutrient depletion, acidity, high osmolarity, and preservatives [10,11]. These stresses, linked to food processing, preservation, and storage, exert selective pressure on foodborne pathogens [12]. However, there is relatively limited information regarding the phenotypic heterogeneity, specifically persistence, in antibiotic-resistant bacteria when exposed to food preservative-related stresses. Bacterial persister cells are an emerging concern in microbiological food safety. Recently, persisters have gained renewed attention owing to treatment failures and chronic infections. Persisters, unlike antibiotic-resistant bacterial cells, display transient phenotypic tolerance to antimicrobials and environmental stresses, enabling them to endure harsh conditions. The presence of persisters can cause the potential dissemination of antibiotic resistance within food systems.

The mechanisms underlying persister formation are dependent on exposure to a range of stresses. Notably, various foodborne pathogens can form persister cells when exposed to stresses encountered during food processing and preservation. Therefore, the aim of this study was to evaluate the heterogeneous phenotypic responses of antibiotic-resistant *Salmonella Typhimurium* to food preservative-related stresses, lactic acid and sodium chloride (NaCl). Lactic acid is widely used in the food industry as a natural preservative. The antimicrobial effect of lactic acid is attributed to its undissociated form, leading to intracellular acidification, the disruption of cellular metabolic processes, interference with enzymatic activities, and the denaturation of protein structures [13]. NaCl has been employed as a food preservative through methods such as pickling, curing, and brining, which are unfavorable for bacterial growth [14]. Plasmolysis can reduce water activity, inhibit bacterial growth, and eventually enhance the shelf life of foods [14]. The presence of antibiotic-resistant pathogens within the food chain poses a significant risk to the safety of food products [15].

## 2. Results

### 2.1. Susceptibilities of S. Typhimurium Serially Exposed to Ciprofloxacin and Food Processing-Related Stresses

**Figure 1:**
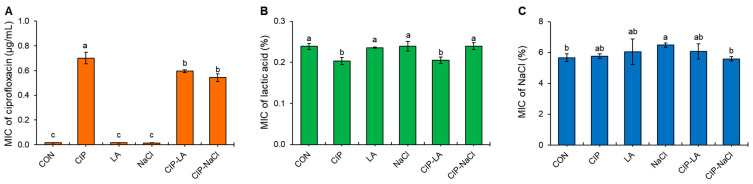
Minimum inhibitory concentrations (MICs) of (**A**) ciprofloxacin (CIP), (**B**) lactic acid (LA), and (**C**) NaCl, against *Salmonella Typhimurium* treated with CIP, LA, NaCl, CIP-LA, and CIP-NaCl. Treatments CIP, LA, NaCl, CIP-LA, and CIP-NaCl indicate pre-exposure to ciprofloxacin (1 µg/mL), 0.2% lactic acid, 6% sodium chloride, ciprofloxacin followed by lactic acid, and ciprofloxacin followed by sodium chloride, respectively. CON represents the untreated control. Different letters (a–c) on the bars are significantly different at *p* < 0.05. Data shown are averages of three biological replicates.

An antimicrobial susceptibility assay was used to evaluate the ciprofloxacin, lactic acid, and NaCl. The susceptibilities of *S. Typhimurium* cells treated with CON, CIP, LA, NaCl, CIP-LA, and CIP-NaCl to ciprofloxacin, lactic acid, and NaCl were evaluated as shown in Figure 1. The MICs of ciprofloxacin in the CIP, CIP-LA, and CIP-NaCl groups were significantly increased to 0.70, 0.59, and 0.54 µg/mL, respectively, when compared to the CON group (0.014 µg/mL) (Figure 1A). There was no significant change in the susceptibilities of the LA and NaCl treatments to ciprofloxacin, showing less than 0.02 µg/mL, compared to the CON group. The CIP and CIP-LA groups were more susceptible to lactic acid than the CON group, while the MICs of lactic acid in the LA and NaCl groups were similar to the MIC of lactic acid in the CON group (0.24%) (Figure 1B). The NaCl susceptibilities of all treatments were not changed, with the exception of the NaCl treatment, which showed an increase in NaCl resistance (Figure 1C).

The MBCs and EECs of ciprofloxacin, lactic acid, and NaCl against *S. Typhimurium* cells treated with CON, CIP, LA, NaCl, CIP-LA, and CIP-NaCl were extensively determined to evaluate the bactericidal and Eagle effects, respectively (Table 1). The highest MBC of ciprofloxacin was observed in the CIP-LA group (4.0 µg/mL), followed by the CIP (2.0 µg/mL) and CIP-NaCl groups (1.0 µg/mL) (Table 1). However, the MBCs of lactic acid and NaCl against all treatments were not noticeably changed compared to the CON group. The Eagle phenomena were observed in the CIP, CIP-LA, and CIP-NaCl groups in the presence of ciprofloxacin, showing EECs of 8.0, 8.0, and 16.0 µg/mL, respectively. The EECs of lactic acid and NaCl were not detected in any treatment (CON, CIP, LA, NaCl, CIP-LA, and CIP-NaCl).

### 2.2. Phenotypic Heterogeneity of S. Typhimurium Serially Exposed to Ciprofloxacin and Food Processing-Related Stresses

**Figure 2:**
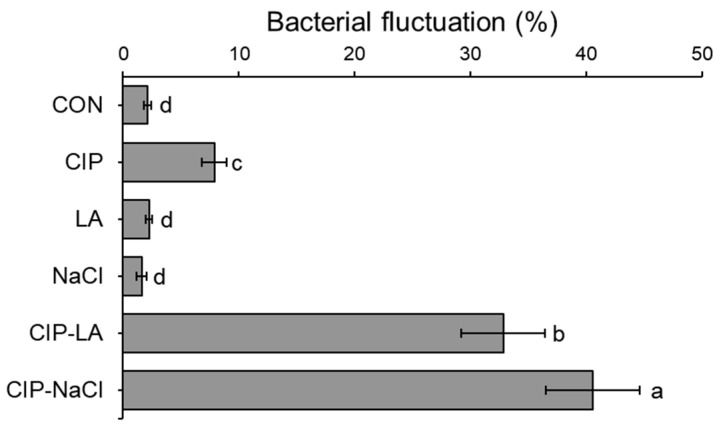
Fluctuation analysis of *Salmonella Typhimurium* treated with ciprofloxacin (CIP), lactic acid (LA), NaCl, CIP-LA, and CIP-NaCl. Treatments CIP, LA, NaCl, CIP-LA, and CIP-NaCl indicate pre-exposure to ciprofloxacin (1 µg/mL), 0.2% lactic acid, 6% sodium chloride, ciprofloxacin followed by lactic acid, and ciprofloxacin followed by sodium chloride, respectively. CON represents the untreated control. Bacterial fluctuations are expressed as coefficient of variance (%). Different letters on the bars (a–d) are significantly different at *p* < 0.05. Data shown are averages of three biological replicates.

**Figure 3:**
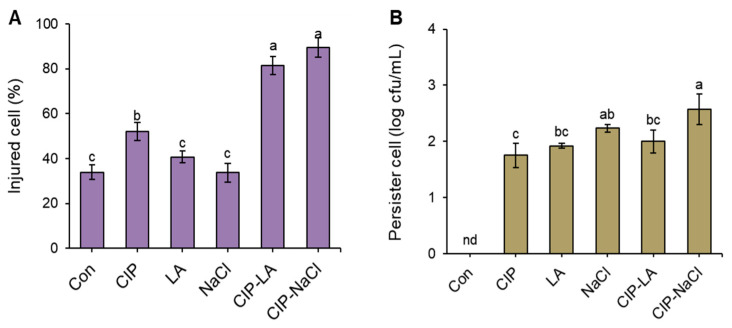
Injured (**A**) and persistent (**B**) *Salmonella Typhimurium* cells induced by ciprofloxacin (CIP), lactic acid (LA), NaCl, CIP-LA, and CIP-NaCl. Treatments CIP, LA, NaCl, CIP-LA, and CIP-NaCl indicate pre-exposure to ciprofloxacin (1 µg/mL), 0.2% lactic acid, 6% sodium chloride, ciprofloxacin followed by lactic acid, and ciprofloxacin followed by sodium chloride, respectively. CON represents the untreated control. Different letters on the bars (a–c) are significantly different at *p* < 0.05. “nd” denotes not detected. Data shown are averages of three biological replicates.

**Figure 4:**
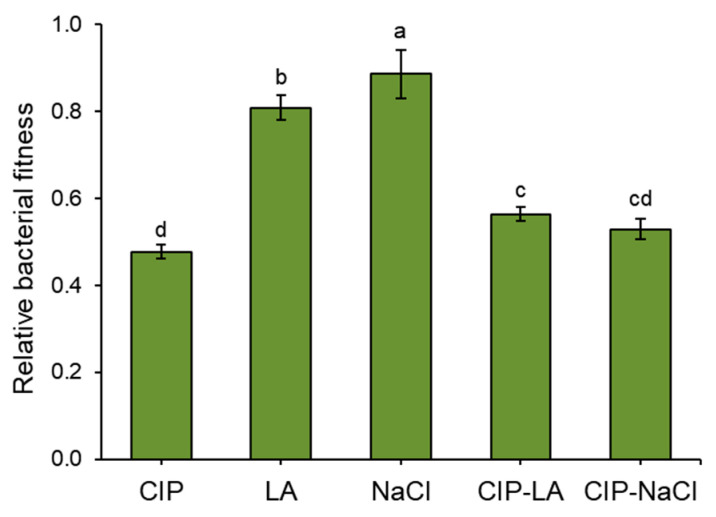
Relative fitness of *Salmonella Typhimurium* treated with ciprofloxacin (CIP), lactic acid (LA), NaCl, CIP-LA, and CIP-NaCl. Treatments CIP, LA, NaCl, CIP-LA, and CIP-NaCl indicate pre-exposure to ciprofloxacin (1 µg/mL), 0.2% lactic acid, 6% sodium chloride, ciprofloxacin followed by lactic acid, and ciprofloxacin followed by sodium chloride, respectively. CON represents the untreated control. Different letters on the bars (a–d) are significantly different at *p* < 0.05. Data shown are averages of three biological replicates.

The bacterial heterogeneity of *S. Typhimurium* cells treated with CON, CIP, LA, NaCl, CIP-LA, and CIP-NaCl was evaluated using the bacterial fluctuation (Figure 2), cell injury (Figure 3A), and persistence (Figure 3B). A bacterial fluctuation assay was used to determine the cell variability. The highest bacterial fluctuation was observed for CIP-NaCl (41%), followed by CIP-LA (33%) (Figure 2). The agar overlay assay was used to estimate the injured cells. The proportions of injured cells were significantly increased in the *S. Typhimurium* cells treated with CIP-LA and CIP-NaCl, showing more than 80% (Figure 3A). But the LA and NaCl treatments showed no significant difference in the proportions of injured cells when compared to the CON group. The persister cells were estimated at extreme concentrations of gentamicin (10 × MIC). Persister cells were not induced in the CON group, showing less than the detection limit of 1.3 log cfu/mL (Figure 3B). The highest persister cells were induced by the CIP-NaCl treatment (>2 log cfu/mL). *S. Typhimurium* cells treated with CON, CIP, LA, NaCl, CIP-LA, and CIP-NaCl were cultured in antibiotic-free media to evaluate their relative fitness of resistance (Figure 4).

**Figure 5:**
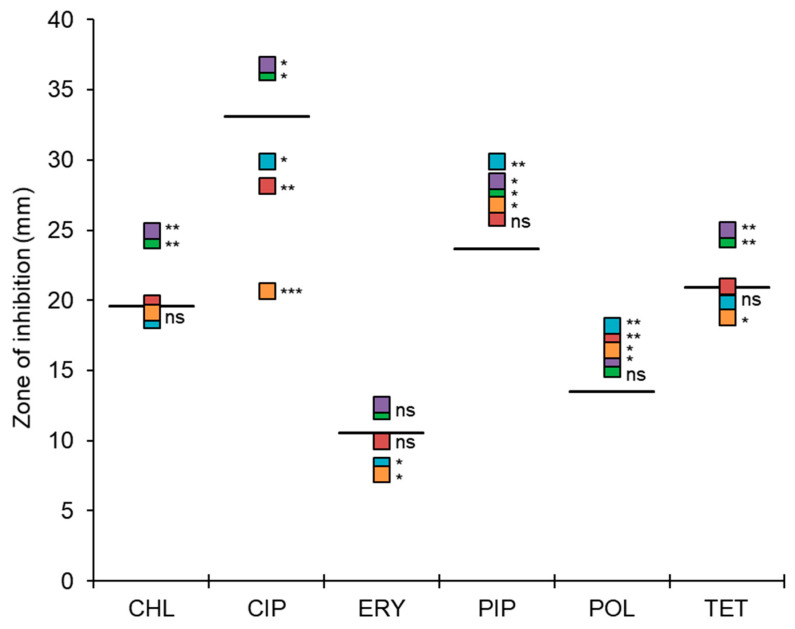
Cross-resistance of *Salmonella Typhimurium* treated with ciprofloxacin (CIP; ■), lactic acid (LA; ■), NaCl (■), CIP-LA (■), and CIP-NaCl (■) to chloramphenicol (CHL), ciprofloxacin (CIP), erythromycin (ERY), piperacillin (PIP) polymyxin B (POL), and tetracycline (TET). ns denotes no significant difference. Treatments, CIP, LA, NaCl, CIP-LA, and CIP-NaCl, indicate pre-exposure to ciprofloxacin (1 µg/mL), 0.2% lactic acid, 6% sodium chloride, ciprofloxacin followed by lactic acid, and ciprofloxacin followed by sodium chloride, respectively. *, **, and *** are significantly different from the CON at *p* < 0.05, *p* < 0.01, and *p* < 0.001, respectively. CON (––) represents the untreated control. Data shown are averages of three biological replicates.

The highest relative fitness levels were observed for the LA and NaCl treatments, suggesting a decrease in the fitness cost. The CIP, CIP-LA, and CIP-NaCl treatments had low relative fitness levels of 0.48, 0.56, and 0.53, respectively. The disk diffusion assay was used to determine the cross-resistance. The susceptibilities of *S. Typhimurium* cells treated with CON, CIP, LA, NaCl, CIP-LA, and CIP-NaCl to chloramphenicol (CHL), ciprofloxacin (CIP), erythromycin (ERY), piperacillin (PIP), polymyxin B (POL), and tetracycline (TET) were determined to evaluate the development of antibiotic cross-resistance (Figure 5) The CIP-exposed treatments, CIP, CIP-LA, and CIP-NaCl, showed significant resistance to the same CIP compared to CON. *S. Typhimurium* cells treated with CIP-LA and CIP-NaCl were cross-resistant to ERY. The cross-resistance of *S. Typhimurium* to TET was observed for the CIP-NaCl treatment. However, the *S. Typhimurium* cells treated with the LA and NaCl treatments showed enhanced susceptibilities to CHL, CIP, PIP, and TET.

## 3. Discussion

The emergence of antibiotic resistance presents a substantial challenge in healthcare and food safety due to the difficulty of effectively controlling bacterial infections and food contaminations. During food processing and preservation, antibiotic-resistant bacteria can frequently encounter adverse conditions, such as osmotic and acid stresses, enabling them to transition into various metabolic states [16]. However, the phenotypic heterogeneity of antibiotic-resistant bacteria has not been well studied in food processing and preservation environments. Specifically, persister cells are relatively less investigated and even underestimated due to the lack of information about their potential food safety risks. The presence of persister cells in the food chain is a significant consideration when assessing microbiological food safety and controlling the tolerant subpopulation. The persister cells are affected by the inhibition of replication, transcription, and translation. Ironically, proteins are needed to maintain the bacterial persistence that is not directly affected by the inhibition of nucleic acid and protein synthesis. The phenotypic heterogeneity found in dormant cells is a result of bet-hedging strategies designed to increase survival and reproduction rates in the face of unpredictable environmental stresses. The mechanisms driving persister formation are diverse and depend on factors such as antibiotic class, growth phase, and cell type. Therefore, understanding these mechanisms is crucial for controlling persister cells in food environments and developing novel anti-persister agents. Therefore, this study could provide useful information for understanding the phenotypic heterogeneity of antibiotic-resistant foodborne pathogens when exposed to food preservative-related stresses.

The pre-exposure to ciprofloxacin (CIP) considerably increased the resistance to the same antibiotic, while LA and NaCl did not induce or slightly increased the resistance to lactic acid and NaCl, respectively (Figure 1). These results suggest that bacteria develop resistance when repeatedly exposed to the same stresses that exert a strong selective pressure [17]. Ciprofloxacin, a widely used fluoroquinolone antibiotic, inhibits DNA gyrase and topoisomerase IV, leading to the disruption of DNA replication and transcription [18]. However, prolonged ciprofloxacin exposure can exert selective pressure, leading to the evolution of antibiotic-resistant mutants [19]. CIP-LA, CIP, and CIP-NaCl had enhanced resistance to ciprofloxacin (MIC ≥ 1 µg/mL) and also induced the Eagle effect (EEC ≥ 8 µg/mL) (Table 1). This result implies that antibiotic-resistant foodborne pathogens exhibit cross-resistance to food preservative-related stresses. The Eagle effect describes the ability of bacteria to survive over bactericidal concentrations of antibiotics due to the presence of slow-growing cells, which results in reduced antibiotic target sites and enhanced antibiotic resistance [20,21]. The Eagle phenomenon and persistence share similar phenotypic changes in response to antibiotics [22]. This is in good agreement with a previous report showing that antibiotic-induced persister cells had higher MBCs than untreated control cells [23].

The treatments exposed to ciprofloxacin (CIP, CIP-LA, CIP-NaCl) exhibited higher fluctuation rates compared to the CON, LA, and NaCl groups (Figure 2). Bacterial fluctuation can result from various factors, including genetic mutations, phenotypic variation, and selection pressures, leading to the emergence of antibiotic-resistant mutants [24,25]. Bacteria may undergo phenotypic changes that enhance their survival in the presence of ciprofloxacin, but these changes may not be sustained in the absence of the antibiotic. CIP-LA and CIP-NaCl induced reversible cell injury (Figure 3A), corresponding to the highest bacterial fluctuations, as shown in Figure 2. Persister cells were induced by all treatments, except for CON (Figure 3B). This implies that antibiotic-resistant bacteria, when exposed to food preservative-related stresses, induce phenotypic heterogeneity within a bacterial population. The presence of bacterial persistence within a population can be a contributing factor to antibiotic resistance in bacteria [26]. Bacterial persistence is characterized by bistability, multistability, stochastic switching, or phenotypic heterogeneity [27]. In this phenomenon, normal wild-type bacterial cells can undergo a phenotypic transition into persister cells, which consist of a small subpopulation of dormant cells [28]. Persister cells have the ability to sense the presence of antibiotics, enabling them to revert to a wild-type state when exposed to favorable conditions [29].

The persistence of foodborne pathogens has been observed in food processing-related stresses [30]. Persisters can emerge in response to various conditions, such as nutrient depletion, toxin overexpression, and metabolic shift [31,32,33]. Stresses associated with food processing can trigger the formation of persisters within the food chain [34]. Slow-growing bacteria can transition into a persister state, temporarily gaining resistance to antimicrobials that typically target actively growing bacteria [35,36]. The presence of growth-arrested persister cells has raised concerns in food industry because they pose challenges in terms of effective treatment [37,38]. Persisters can lead to prolonged and recurrent contaminations, ultimately resulting in failure of antimicrobial treatments. Moreover, food safety can be underestimated due to the presence of persisters cells within the food chain [39]. Persister cells revert to their intrinsic phenotype after growth in the absence of stresses, referred to as resuscitation [40,41]. In contrast, dose-dependent persisters exhibit transient resistance during the first antimicrobial exposure but become susceptible to subsequent antimicrobial treatment [21]. The long-term presence of persister cells in food products poses significant safety risks [30,42]. Consequently, persister cells can be a major contributor to microbial contamination, eventually leading to failure in the control of microbial food safety. Although not fully understood, this phenomenon can be observed during food processing and preservation.

Low relative fitness levels were observed in the CIP, CIP-LA, and CIP-NaCl treatments (Figure 4). This suggests that antibiotic-resistant bacteria in the absence of antibiotics may not restore a fitness cost as much as wild-type cells. In the absence of antibiotics, antibiotic-susceptible bacteria outcompete their antibiotic-resistant counterparts that require extra energy to sustain their mechanisms of antibiotic resistance [25]. Antibiotic-resistant bacteria pay a fitness cost for adapting to new environments through mutations or alterations in cellular metabolisms [43]. The chromosomal mutations that confer antibiotic resistance in bacteria are responsible for an increase in fitness costs [44]. The development of antibiotic resistance frequently imposes a burden on the overall fitness of bacteria, influencing survival, competition, growth, and virulence [45]. Understanding the dynamics of antibiotic resistance is significantly influenced by the concept of fitness cost. This concept refers to the potential disadvantages or drawbacks that bacteria may face as a result of developing and maintaining resistance mechanisms. The extent of this fitness cost is intricately linked to both the evolutionary process of resistance development and the sustainability of the established resistance [46].

The CIP, CIP-LA, and CIP-NaCl groups were resistant to ciprofloxacin, the CIP-LA and CIP-NaCl groups were cross-resistant to ERY, and the CIP-NaCl group was cross-resistant to TET (Figure 5). Cross-resistance is mainly due to the antibiotic selection pressure [47]. Antibiotic cross-resistance, where exposure to one antibiotic leads to resistance against others, is a significant concern in the context of antibiotic therapy. Cross-resistance occurs when resistance mechanisms developed in response to one antibiotic confer protection against other antibiotics with similar targets or modes of action [48]. The exposure to ciprofloxacin can confer resistance to other classes of antibiotics, such as β-lactams, aminoglycosides, and macrolides [49]. However, LA and NaCl were susceptible to CHL, CIP, PIP, and TET. This result is known as negative cross-resistance or collateral susceptibility that restores antimicrobial activity and reduces antibiotic resistance [50]. The acquisition of resistance to one antibiotic may result in the increased susceptibility to another antibiotic [51].

## 4. Materials and Methods

### 4.1. Bacterial Strain and Culture Condition

The strain of *Salmonella enterica* subsp. *enterica* serovar Typhimurium ATCC 19585, purchased from the American Type Culture Collection (ATCC, Manassas, VA, USA), was cultured in trypticase soy broth (TSB; BD; Becton, Dickinson and Co., Sparks, MD, USA) for 18 h at 37 °C. The activated cells were washed twice by centrifugation at 6000× *g* for 10 min at 4 °C in phosphate-buffered saline (PBS; pH 7.2). The collected cells were resuspended in PBS to obtain approximately 10^8^ cfu/mL and properly diluted prior to analysis.

### 4.2. Antimicrobial Susceptibility Assay

The *Salmonella Typhimurium* cells (10^6^ cfu/mL) were pre-exposed to ciprofloxacin from 0.03 to 1 µg/mL (CIP), 0.2% lactic acid (LA), 6% NaCl, CIP followed by LA (CIP-LA), and CIP followed by NaCl (CIP-NaCl). The ciprofloxacin-resistant *S. Typhimurium* was induced according to a stepwise selection assay [52]. In brief, the wild-type strain of *S. Typhimurium* ATCC 19585 was consecutively cultured in TSB and TSA by increasing the concentrations of ciprofloxacin up to 1 µg/mL. Once the ciprofloxacin-resistant *S. Typhimurium* cells were induced after several passages, the stability of the acquired resistance in *S. Typhimurium* was confirmed by comparing the ciprofloxacin susceptibility of the wild-type with that of the induced *S. Typhimurium*. The treatment concentrations of LA and NaCl were determined based on the minimum inhibitory concentrations (MICs). The susceptibilities of the untreated control (CON) and the treatments (CIP, LA, NaCl, CIP-LA, and CIP-NaCl) to ciprofloxacin, lactic acid, and NaCl were evaluated according to the Clinical Laboratory Standards Institute (CLSI) procedure. Ciprofloxacin, lactic acid, and NaCl stock solutions were diluted from 16 µg/mL, 1%, and 10%, respectively, with fresh TSB in 96-well plates (BD Falcon, San Jose, CA, USA). Approximately 10^6^ cfu/mL were inoculated in 96-well plates containing stock solutions and incubated at 37 °C for 18–72 h to determine the minimum inhibitory concentrations (MICs) and Eagle effect concentrations (EECs). The cultures (100 µL) in wells with no visible growth were further cultured in fresh TSB (5 mL) to determine minimum bactericidal concentrations (MBCs). After 24 h of incubation at 37 °C, the MBC was the concentration at which there was no visible growth in the subculture.

### 4.3. Bacterial Fluctuation Assay

The fluctuation assay was used to determine the cell variability in the CON, CIP, LA, NaCl, CIP-LA, and CIP-NaCl treatments. The treated cells (10 cfu/mL each) were distributed into 96-well plates and incubated at 37 °C for 24 h. The bacterial growths at 24 h were measured at an optical density at 600 nm. The growth fluctuations were estimated by using a coefficient of variation (CV).

### 4.4. Agar Overlay Assay

The cells injured by treatments (CON, CIP, LA, NaCl, CIP-LA, and CIP-NaCl) were determined using an agar overlay assay with minor modification [53]. Each treatment was diluted to the same cell counts ranging from 100 to 200. Aliquots from each dilution were plated on TSA as the nonselective medium and xylose lysine deoxycholate (XLD) agar as the selective medium. After 3 h of incubation at 37 °C, TSA with 0.6% yeast extract (TSAYE) was overlaid, and they were further incubated for 24–48 h. Differences in the counts on the TSA and XLD agar were used to evaluate the recovery behavior of cells in the CON, CIP, LA, NaCl, CIP-LA, and CIP-NaCl treatments.

### 4.5. Estimation of Persister Cells

The persister cells induced by the CON, CIP, LA, NaCl, CIP-LA, and CIP-NaCl treatments were estimated by exposing them to high concentrations of antibiotics [54]. In brief, *S. Typhimurium* cells (10^6^ cfu/mL each) in the different treatments were incubated in TSB containing 10 × MIC of gentamicin (40 µg/mL) for 5 h at 37 °C. The cultures were diluted (1:10) with PBS and plated on TSA using the Autoplate^®^ Spiral Plating System (Spiral Biotech Inc., Norwood, MA, USA). The plates were incubated at 37 °C for 24–48 h and counted using the QCount^®^ Colony Counter (Spiral Biotech Inc., Norwood, MA, USA).

### 4.6. Relative Fitness Determination

The *S. Typhimurium* cells treated with CON, CIP, LA, NaCl, CIP-LA, and CIP-NaCl were harvested by centrifugation at 5000× *g* for 10 min at 4 °C and diluted to 10^2^ cfu/mL. The dilutes were distributed into a 96-well plate and cultured at 37 °C for 24 h in antibiotic-free fresh TSB. The relative fitness was expressed as the ratio of the growths of treatments (CIP, LA, NaCl, CIP-LA, and CIP-NaCl) to the growth of the untreated control (CON) [55].

### 4.7. Disk Diffusion Susceptibility Test

The cross-resistance of the *S. Typhimurium* cells in the CON, CIP, LA, NaCl, CIP-LA, and CIP-NaCl treatments was evaluated using a disk diffusion assay. The treated cells were evenly spread onto the surface of Mueller–Hinton (MH) agar, and antibiotic discs (Oxoid Ltd., Hampshire, UK), including chloramphenicol (CHL; 30 μg), ciprofloxacin (CIP; 5 μg), erythromycin (ERY; 30 μg), piperacillin (PIP; 30 μg), polymyxin B (POL; 30 μg), and tetracycline (TET; 30 μg), were placed on the agar. The MH agar plates were incubated at 37 °C for 24 h, and the diameters of the clear zone were measured using a digital vernier caliper (The L.S. Starrett Co., Athol, MA, USA).

### 4.8. Statistical Analysis

All assays were conducted in three biological replicates. Statistical Analysis System (SAS Institute, Inc., NC, USA) version 9.4 software was used to analyze the data with the general linear model (GLM) and Fisher’s least significant difference (LSD) procedures and to determine significant differences at *p* < 0.05, *p* < 0.01, and *p* < 0.001.

## 5. Conclusions

In conclusion, this study emphasizes the significance of the phenotypic heterogeneity of antibiotic-resistant *S. Typhimurium* cells when exposed to food preservative-related stresses. The most significant findings in this study are that antibiotic-resistant *S. Typhimurium* cells, when successively exposed to food preservative-related stresses, such as CIP-LA and CIP-NaCl, induced various phenotypic changes in bacterial fluctuation, cell damage, persistence, and cross-resistance. Interestingly, persisters exhibited remarkable growth at extremely lethal concentrations (>MIC), a phenomenon known as the Eagle effect. The pre-exposure to ciprofloxacin can significantly influence bacterial fluctuation, leading to cross-resistance and collateral sensitivity, which are essential for designing effective antimicrobial regimens in food processing and preservation. The enhanced phenotypic diversity observed in pre-exposed populations has direct implications for antibiotic resistance. Unlike antibiotic-resistant counterparts, persister cells exhibit a unique ability to endure extreme antibiotic concentrations, posing potential risks in food systems. Due to their vague nature and limited attention, understanding the mechanisms of persister cell formation has become an essential part in the food industry. Therefore, this study sheds light on the risks associated with bacterial persistence of antibiotic-resistant bacteria when exposed to food preservative-related stresses. To clearly address these challenges, future research should focus on unraveling the intricate mechanisms of persister formation, identifying reliable biomarkers, and developing innovative anti-persister strategies in food environments.

## Figures and Tables

**Table 1:** Minimum bactericidal concentrations (MBCs) and Eagle effect concentrations (EECs) of ciprofloxacin (CIP), lactic acid (LA), and NaCl against *Salmonella Typhimurium* treated with CIP, LA, NaCl, CIP-LA, and CIP-NaCl.

Treatment *	MBC	EEC	MBC	EEC	MBC	EEC
	Ciprofloxacin		Lactic Acid		NaCl	
CON	0.03	nd **	0.40	nd	10.0	10.0
CIP	2.0	8.0	0.40	nd	10.0	10.0
LA	0.03	nd	0.40	nd	10.0	10.0
NaCl	0.03	nd	0.30	nd	9.0	9.0
CIP-LA	4.0	8.0	0.40	nd	10.0	10.0
CIP-NaCl	1.0	16.0	0.30	nd	9.0	9.0

* Treatments CIP, LA, NaCl, CIP-LA, and CIP-NaCl indicate pre-exposure to ciprofloxacin (1 µg/mL), 0.2% lactic acid, 6% sodium chloride, ciprofloxacin followed by lactic acid, and ciprofloxacin followed by sodium chloride, respectively. CON represents the untreated control. ** nd denotes not detected.

## Data Availability

Data are contained within the article.
